# Comparison of surgical approaches and outcome for symptomatic pineal cysts: microscopic/endoscopic fenestration vs. stereotactic catheter implantation

**DOI:** 10.1007/s00701-025-06445-3

**Published:** 2025-01-31

**Authors:** Michael Schmutzer-Sondergeld, Aylin Gencer, Tristan Schmidlechner, Hanna Zimmermann, Sebastian Niedermeyer, Sophie Katzendobler, Veit M. Stoecklein, Thomas Liebig, Christian Schichor, Niklas Thon

**Affiliations:** 1 Department of Neurosurgery, LMU University Hospital, Ludwig-Maximilians-University Munich, Marchioninistrasse 15, 81377 Munich, Germany; 2 Institute of Diagnostic and Interventional Neuroradiology, LMU University Hospital, LMU Munich, Marchioninistrasse 15, 81377 Munich, Germany; 3 German Cancer Consortium (DKTK), Partner Site Munich, and German Cancer Research Center (DKFZ), Heidelberg, Germany

**Keywords:** Pineal cysts, Microsurgery, Endoscopy, Stereotaxy, Evans index, FOHR

## Abstract

**Purpose:**

Treatment strategies for space-occupying/symptomatic pineal cysts (PC) are still up for debate. In this study we present PC management, outcome data and risk factors for recurrence after surgery, focusing on microscopic/endoscopic procedures vs. stereotactic catheter implantation as alternative treatment concept to permanently drain PC into ventricles/cisterns.

**Methods:**

This monocentric retrospective analysis included clinical data from all consecutive PC patients treated surgically between 2000 and 2022. Postoperative neurological and functional outcomes, along with perioperative complications, as well as time to PC recurrence and MR-morphological data were evaluated.

**Results:**

39 patients (median age 32.6 years, range: 5.1–71.6 years) were analyzed. Main presenting symptoms were headaches, visual impairment, and epileptic seizures. In 18 patients (46.2%) an enlarged ventricular system was preoperatively found with 7 patients (18.0%) suffering from occlusive hydrocephalus. 14 patients underwent microscopic/endosocopic surgery, in 25 cases stereotaxy was preferred. No complication was seen in the microsurgery/endoscopy group compared to one intracystic postoperative bleeding (2.6%) and two CSF leaks (5.1%) after stereotaxy (*p* = 0.5). Overall, clinical improvement and significant cyst volume reduction (*p* < 0.0001) was seen in all patients. Recurrent PC were seen in 23.1%, independent of surgical procedure (*p* = 0.2). In cases of recurrence, TTR was 25.2 ± 31.2 months. Male gender (*p* = 0.01), longer surgery time (*p* = 0.03) and preoperatively increased Evans index (EI) (*p* = 0.007) were significant risk factors for PC recurrence in multivariate analysis.

**Conclusion:**

In patients suffering from PC, microsurgical and stereotactic approaches can improve clinical symptoms at low procedural risk, with equal extent of volume reduction. However, preoperative ventricular enlargement and EI values should be considered for optimal treatment planning to reduce recurrence.

**Supplementary Information:**

The online version contains supplementary material available at 10.1007/s00701-025-06445-3.

## Introduction

The majority (80%) of all pineal cysts (PC) are small benign lesions in the pineal gland with a diameter of less than 10 mm and are usually asymptomatic [6, 14, 19, 31, 37, 41]. Due to the increasing rate of cerebral imaging scans (cerebral computed tomography (cCT) and cerebral magnetic resonance imaging (cMRI)), the incidences of these asymptomatic PC has risen over the last decades [6, 14, 19, 31, 37, 41]. If the PC volume exceeds, these cysts can block the aqueduct and lead to a cerebrospinal fluid circulation disorder in the form of a hydrocephalus occlusus and/or compress neighboring neuronal anatomical structures such as the tectum. Clinically, patients often develop headaches, seizures or even persisting neurological deficits like eye movement disorders. These changes occur more frequently in early life, so that most PC are diagnosed in adolescence and young adults. However, they can also occur later in life [31].

Asymptomatic patients are usually not operated on but monitored at interval using cMRI [15, 20, 25, 40]. However, if symptoms, including atypical complaints, such as epileptic seizures or vertigo, develop during the course of the disease, the indication for surgery should be re-evaluated. In here, the indication for treatment is largely determined by the compatibility of the main clinical symptoms with PC and after exclusion of other possible neurological causes [20, 35]. Image morphological features such as ruptures or hemorrhages may make urgent treatment necessary, but may also cause symptoms to recede [14, 15, 17, 20, 24–26, 37, 40–42].

Various approaches exist for the surgical treatment of cystic lesions of the pineal gland, such as open or endoscopic cyst fenestration or resection, endoscopic third ventriculocisternostomy (ETV), stereotactic cyst puncture or stereotactic implantation of an (internal) shunt for the treatment of concomitant hydrocephalus occlusus in aqueductal stenosis. The choice of surgical procedure is determined by cyst size, the presence of hydrocephalus and neurological deficits but also by the availability and experience of the treating neurosurgical center with regard to alternative treatment methods. However, there is very little prospective data on the optimal surgical approach [15, 20, 23, 25]. Furthermore, there are no studies that present postoperative symptom management according to the individual surgical approaches.

The aim of this study is to use a retrospectively collected data set to analyze and compare patients who underwent various surgical approaches for PC at our department between 2002 and 2022 and were followed up postoperatively and to make some recommendations for treatment.

## Materials and methods

### Patient population

After approval from the institutional review board of the Ludwig-Maximilians-University Munich (reference number 23–0621), the patient database of the department of neurosurgery was searched for all patients who underwent any surgical treatment of newly diagnosed PC between January 2000 and December 2022. Indications for surgical therapy were refractory visual disturbances, progressive headaches, or new-onset neurologic deficits such as epileptic seizures or a reduction of vigilance after obtaining a cMRI that confirms the presence of PC. In patients with MR-morphological evidence of a PC, non-specific, refractory progressive headache symptoms and unsuccessful conservative treatment attempts, indication for surgery was given after excluding all other possible causes. Clinical and diagnostic data were collected preoperatively and at routine follow-up evaluations (normally at 3 months, 12 months, 24 months or later). Functional outcome analyses referred to preoperatively obtained data. All patients and/or their parents gave informed consent before surgical treatment.

### Magnetic resonance imaging

According to the standard in-house protocol, the preoperative cMRI (1.5- or 3.0-T scanners: Magnetom Symphony, Siemens, Erlangen; Signa HDxt; GE Healthcare, Little Chalfont, United Kingdom) routinely included axial T2-weighted sequences (with slice thickness of 2 mm), 3-dimensional T1-weighted sequences before and after intravenous administration of gadopentetate dimeglumine (0.1 mmol/kg body weight; Magnevist; Schering Corporation, Kenilworth, NJ), as well as constructive interference in steady-state (CISS) sequences (with slice thickness of 1 mm). Axial, sagittal, and coronal reconstructions were available for each sequence. Volumetric cyst analyses of pre- and postoperative MR images were performed by semi-manual segmentation of pre- and postoperative T2 or CISS and contrast-enhanced (CE) T1 images using a commercially available software tool (SmartBrush®, Elements®, BRAINLAB AG, Munich, Germany). In addition, the Evans index (EI) and fronto-occipital-horn-ratio (FOHR) were determined as markers of pre- and postoperative hydrocephalus/ventriculomegaly on the basis of the cMRI images performed. The EI describes the ratio between the maximum width of the lateral ventricular anterior horns and the maximum inner diameter of the skull on the same slice level [28, 39]. Values greater than 0.3 represent an enlarged ventricular system. The FOHR represents the ratio of the sum of the maximum widths of the anterior and posterior horns of the lateral ventricles divided by twice the maximum internal diameter [1, 32]. Here too, values greater than 0.37 are associated with a widening of the ventricular system [30]. We also determined the cyst-tectum-splenium ratio (CTS) in all patients pre- and postoperatively. The CTS ratio is an indicator for the filling of the pineal recessus and was determined on the mid-sagittal cMRI as the ratio between PC diameter in the area of the shortest distance between tectum and splenium of the corpus callosum and this distance (tectum – splenium of the corpus callosum) [10, 11, 18, 41].

### Treatment protocol

Surgical interventions included microscopic/endoscopic-assisted microsurgical procedures to fenestrate or resect the cyst wall to establish continuous drainage into the physiologic CSF pathways. Either a frontal, transventricular or supracerebellar, intratentorial approach was chosen, depending on the positional relationship of the cyst to the solid pineal tissue, the tectum, the aqueduct and the deep draining veins. For the frontal transventricular approach, a corticotomy was performed in the middle frontal gyrus on the non-dominant side after a precoronary craniotomy and removal of the bone flap. The pineal region was reached via the foramen of Monroe and the third ventricle. The foramen of Monroe was widened using a transchoroidal approach in order to obtain better access. The supracerebellar infratentorial approach (SCIT) was a paramedian approach for adequate visualization of the pineal region in all included cases. The side of the approach was chosen based on the preoperative cMRI in favor of the less dominant transverse sinus. Cysts were drained either by fenestration into the third ventricle (via frontal transventricular approach) or into the cisterna ambiens (via supracerebellar infratentorial approach) by excision of a small window of the cyst membrane or, if surgically feasible, by performing an extended resection of the cyst membrane.

For endoscopic-assisted microsurgery, neuronavigation was used. Either the Lotta or the little Lotta endoscopes (Karl Storz SE & Co. KG) were used for this purpose.

For stereotactic treatment, an internal shunt catheter was implanted stereotactically to connect the cyst to the ventricular system depending on the individual PC localization and configuration. In our cohort a frontal transventricular trajectory was chosen in all patients, which passed through a lateral ventricular anterior horn, the foramen of Monroe and the third ventricle in order to come to rest in the pineal cyst, so that the cyst fluid is permanently drained into the ventricular system. For this, surgical planning (iPlan stereotaxy; Brainlab, Munich, Germany) was based on a stereotactically localized contrast-enhanced computed tomography (CT) scan (0.6 mm slice thickness) and the preoperative MRI data (T1-weighted without contrast, T2-weighted/CISS sequences, contrast-enhanced magnetic resonance angiography), which were merged with the CT scan. A 1.3 mm diameter catheter (Becker EDMS ventricular catheter; Medtronic Inc, Dublin, Ireland) was stereotactically implanted via a 2 mm burr hole. Additional perforations were added manually to the catheter to achieve optimal up- and downstream drainage. The catheter was fixed extracranially with a hemoclip (Titanium Ligation-Clip, 150 mm length, B Braun, Melsungen, Germany) placed orthogonally on the catheter on the calvaria preventing the catheter from sliding into the brain. Above this, a sponge sealant patch (TachoSil®, Takeda Pharmaceuticals, Konstanz, Germany) was attached for adequate closure and additional fixation [21, 29, 33, 38]. The preferred surgical approach was selected for each individual patient in consensus between experienced neurosurgeons depending on cyst configuration, size, and individual risk assessment.

### Outcome analyses

Surgical results and follow-up (FU) analyses were assessed clinically and by quantitative MRI volume measurements at routine FU intervals. Pre- and postoperative clinical conditions of the patients as well as the preoperative Karnofsky performance score (KPS) were analyzed [7, 16]. Clinical outcome data included perioperative complications and the course of symptoms. In particular, the nonspecific visual impairment caused by the dilated optic nerve sheaths and papilledema should be mentioned here, which does not describe a detectable visual field defect in the neurological examination. An increase in postoperative cyst volume of more than 25% or symptomatic progression of the residual cyst was defined as recurrence. If postoperative imaging did not indicate a residual cyst, any new cystic formation during FU was considered a cyst recurrence.

The functional status of the patients was assessed postoperatively using the Chicago Chiari Outcome Score (CCOS) [2, 3]. This scale has already been described and used by several groups [5, 13, 22] for patients after surgery for PC. The CCOS describes the clinical results in terms of “pain”, “no-pain”, “functionality” and “complications” with scores between 4 and 16 [2, 3].

### Risk assessment

Perioperative morbidity rates were determined according to all documented medical, neurological, and approach-related adverse events. Transient and permanent deficits were differentiated.

### Statistical methods

The reference point of this study was the date of first surgery. Last FU date was December 2022. The primary study endpoint was time to PC recurrence (TTR). Secondary endpoints were outcome metrics including clinical symptoms, MR-morphological analyses, and complications. TTR was analyzed by using the Kaplan–Meier method. To compare the survival curves, the log-rank test was used. Results were tested by using a 2-way analysis of variance test (ANOVA), Student’s t-test and Fisher’s exact test. For risk factor analyses uni- and multivariate tests were conducted. For correlation analyses Pearson´s coefficient* r* was determined. GraphPad PRISM 8.0 software was used for statistical analysis (GraphPad, San Diego, CA, USA). Statistical significance was as assumed at *p* < 0.05.

## Results

### Patient characteristics, preoperative symptoms, and surgical treatment

From a total of 90 patients with pineal cysts presenting in our neurosurgical department, 51 patients were clinically observed for asymptomatic PCs. In 39 cases indication for PC surgery was seen. Of these 25 were female (m:f = 1:1.7). The median age was 32.6 years (range: 5.1–71.6 years). The leading clinical symptoms to confirm the indication for surgery were headache (36/39 patients, 92.3%), nonspecific visual impairment mostly characterized as blurred vision (17/39 patients, 43.6%), Parinaud’s syndrome (3/39 patients, 7.7%), epileptic seizures (9/39 patients, 23.1%), vertigo (6/39 patients, 15.4%), and symptoms of increased intracranial pressure (ICP) (4/39 patients, 10.3%). 18/39 patients (46.2%) had an enlarged ventricular system preoperatively with 7/39 patients (18.0%) suffering from occlusive hydrocephalus. Furthermore, 15 patients (38.5%) showed prominent optic nerve sheaths and 3 patients (7.7%) were found with hemorrhaged PC.

**Fig. 1:**
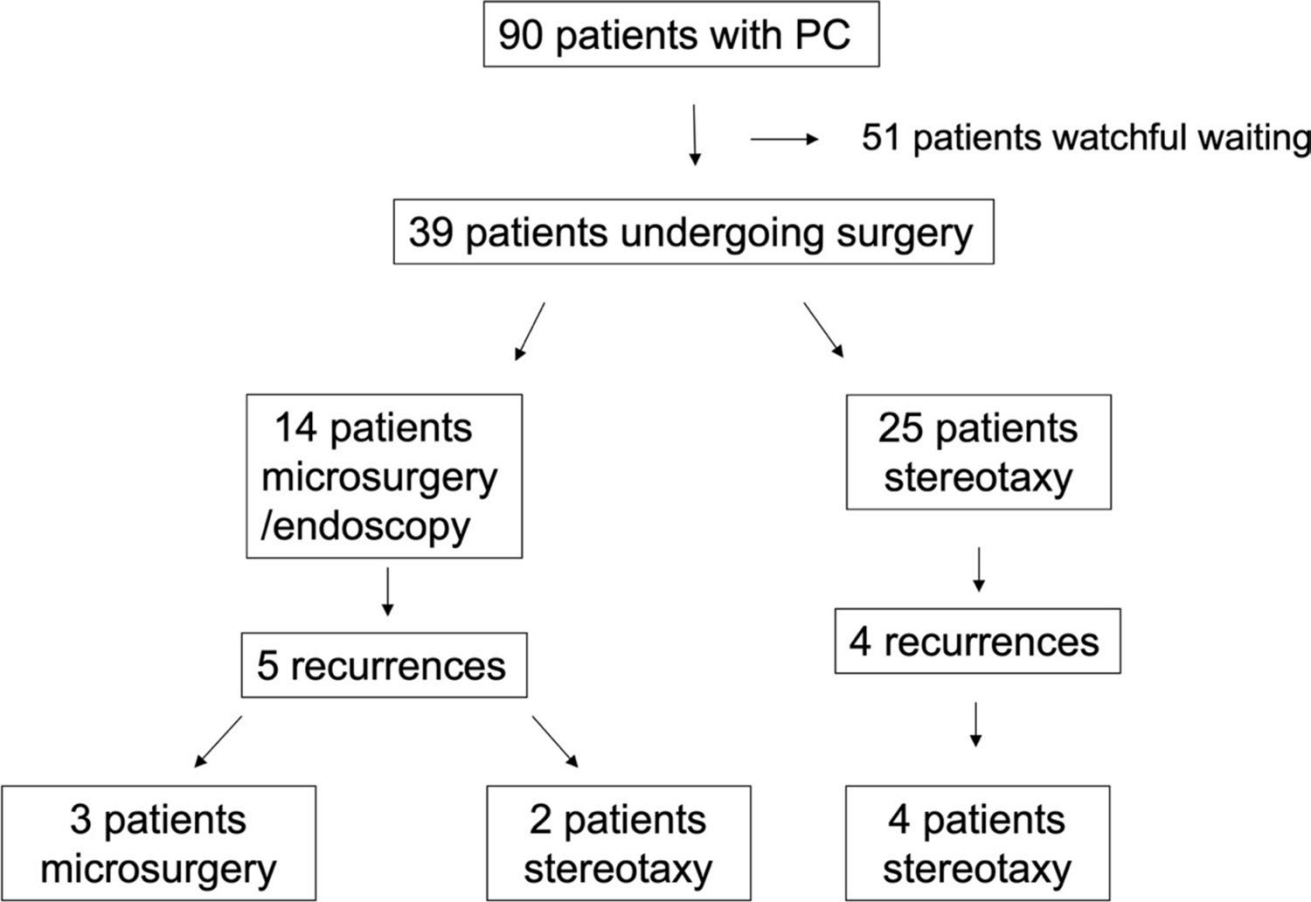
Cohort diagram showing patient selection according to treatment modality

Overall, 14 patients (35.9%) underwent microsurgical (8 patients)/ endoscopical (6 patients) cyst fenestration/ resection and 25 patients received stereotactic implantation of a cystoventricular catheter (see Fig. 1). From a total of 14 patients (8 microsurgical, 6 endoscopic-assisted microsurgical), 8 frontal transventricular approaches (2 microsurgical, 6 endoscopic-assisted microsurgical) and 6 supracerebellar infratentorial approaches were chosen (all microsurgical). In our cohort, 3 pure cyst fenestrations were performed, while 11 extended cyst wall resections were performed. Of the total of 25 patients included with initial stereotactic catheter implantation, a frontal transventricular trajectory was chosen in all patients, which passed through a lateral ventricular anterior horn, the foramen of Monroe and the third ventricle in order to come to rest in the pineal cyst, so that the cyst fluid is permanently drained into the ventricular system. The median duration of symptoms until the first operation was 7.0 months (range: 0.5–80.0 months). There was no difference with respect to the initial presenting symptoms and preferred treatment modality. For details see Table 1.

**Table 1:** Patient characteristics and distribution of preoperative symptoms

Parameters	Microsurgery/Endoscopy	Stereotaxy	*p value*
Patient characteristics			
Total, *n* (%)	14 (35.9)	25 (64.1)	
Sex, *n* (%)			
Male Female	5 (12.8) 9 (23.1)	9 (23.1) 16 (41.0)	0.99
Age, years	38.5 ± 15.6	32.2 ± 16.2	0.2
Karnofsky performance score (KPS), %	86.4 ± 8.4	90.8 ± 6.4	0.07
Pineal cyst hemorrhage, *n* (%)	1 (2.6)	2 (5.1)	0.99
Pineal cyst rupture, *n* (%)	0	0	0.99
Prominent optic sheaths, *n* (%)	4 (10.3)	11 (28.2)	0.5
Parinaud’s syndrome, *n* (%)			
Pre-op Post-op	2 (5.1) 1 (2.6)	0 0	0.1 0.3
Enlarged ventricular system, *n* (%)			
Pre-op Post-op	7 (17.9) 2 (5.1)	11 (28.2) 1 (2.6)	0.7 0.3
Prominent optic sheaths, *n* (%)			
Pre-op Post-op	7 (17.9) 0	8 (20.5) 0	0.3 0.99
Evans index (EI)			
Pre-op Post-op	0.33 ± 0.07 0.29 ± 0.06	0.3 ± 0.07 0.28 ± 0.05	0.2 0.6
Fronto-occipital-horn-ratio (FOHR)			
Pre-op Post-op	0.49 ± 0.09 0.44 ± 0.09	0.47 ± 0.05 0.4 ± 0.07	0.3 0.6
Cyst-tectum-splenium (CTS) ratio			
Pre-op Post-op	0.86 ± 0.06 0.23 ± 0.08	0.87 ± 0.04 0.48 ± 0.12	0.9 **0.001**
Symptom duration until first surgery, months	9.5 ± 3.5	12.9 ± 21.9	0.8
Initial volume (cm^3^)	6.7 ± 4.3	4.6 ± 1.9	0.06
Follow-Up, months	116.0 ± 96.1	64.8 ± 57.1	**0.04**
Time to second surgery (recurrence), months	38.6 ± 36.7	8.5 ± 11.6	0.2
Recurrences, *n* (%)	5 (12.8)	4 (10.3)	0.2
Total number of surgeries, *n* (%)	1.4 ± 0.5	1.2 ± 0.4	0.3
Incision to suture time (min)	166.4 ± 82.1	48.4 ± 7.4	** < 0.0001**
*Symptoms*			
Symptoms pre-op, *n* (%)			
None Paresis Sensory disturbance Headache Epileptic seizures Vertigo Coordination disturbance Increased ICP Cranial nerve symptoms Visual impairment Mnestic disorder	0 1 (2.6) 0 14 (35.9) 2 (5.1) 3 (7.7) 0 1 (2.6) 0 8 (20.5) 0	0 0 0 22 (56.4) 7 (17.9) 3 (7.7) 0 3 (7.7) 0 9 (23.1) 1 (2.6)	0.99 0.3 0.99 0.99 0.4 0.6 0.99 0.99 0.99 0.3 0.99

### Outcome

After surgical therapy, the overall cohort showed a significant improvement of preoperative headaches (*p* < 0.0001), epileptic seizures (*p* = 0.002) and visual deficiencies (*p* = 0.0002). Furthermore, PC-associated dilatated ventricles (*p* = 0.0002), and prominent optic nerve sheaths were improved (*p* < 0.0001) regardless of the type of surgical approach (Table 2). There was no significant difference in the improvement of the individual preoperative symptoms dependent of the surgical modality.

**Table 2:** Distribution of patients` symptoms pre- and postoperatively

Variable	Preoperative *n* (%)	Postoperative *n* (%)	*p value*
Characteristic			
Enlarged ventricular system Parinaud’s syndrome Prominent optic sheaths	18/39 (46.2) 2/39 (5.1) 15/39 (38.5)	3/39 (7.7) 1/39 (2.6) 0	**0.0002** 0.99 ** < 0.0001**
Symptoms			
None Paresis Sensory disturbance Headache Epileptic seizures Vertigo Coordination disturbance Increased ICP Cranial nerve symptoms Visual impairment Mnestic disorder	0 1/39 (2.6) 0 36/39 (92.3) 9/39 (23.1) 6/39 (15.4) 0 4/39 (10.3) 0 17/39 (43.6) 1/39 (2.6)	34/39 (87.2) 0 0 3/39 (7.7) 0 1/39 (2.6) 0 0 0 1/39 (2.6) 0	** < 0.0001** 0.99 0.99 ** < 0.0001** **0.002** 0.1 0.99 0.99 0.99 **0.0002** 0.99

The CCOS in the overall cohort showed a mean value of 14.8 ± 1.6 (range 10–16). Patients after microscopical/endoscopical cyst surgery had a mean value of 14.4 ± 1.4 and after stereotactic catheter implantation of 15.0 ± 1.8 (*p* = 0.3).

**Fig. 2:**
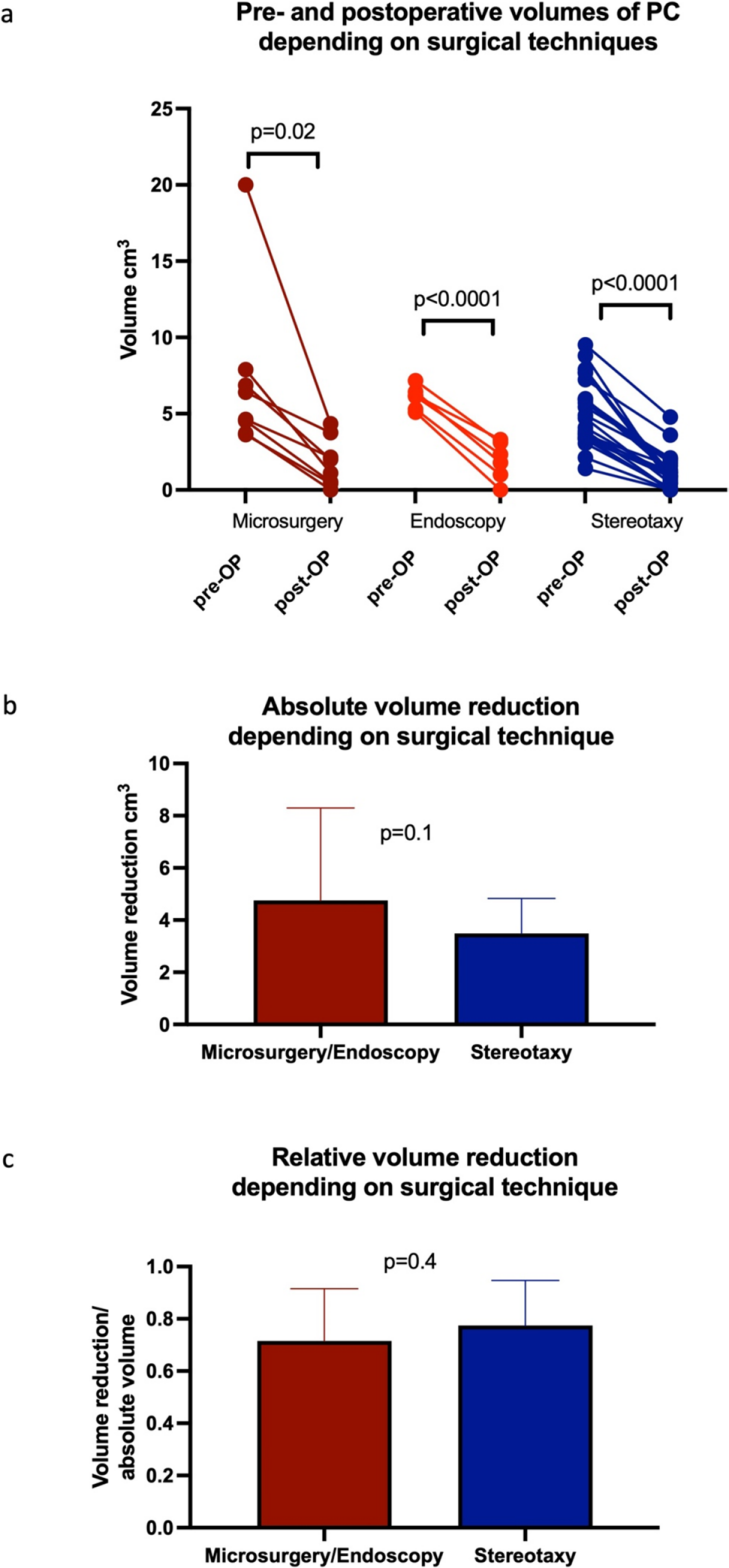
Pre- and postoperative PC volume depending on surgical techniques: microsurgery (*p* = 0.02), endoscopical-assisted microsurgery (*p* < 0.0001) and stereotactic catheter implantation (*p* < 0.0001) in the overall patient cohort (**a**) and absolute (*p* = 0.1, **b**) and relative (*p* = 0.4, **c**) volume reduction depending on surgical treatments microscopic/endoscopic PC wall fenestration and stereotactic implantation of an internal shunt catheter

**Fig. 3:**
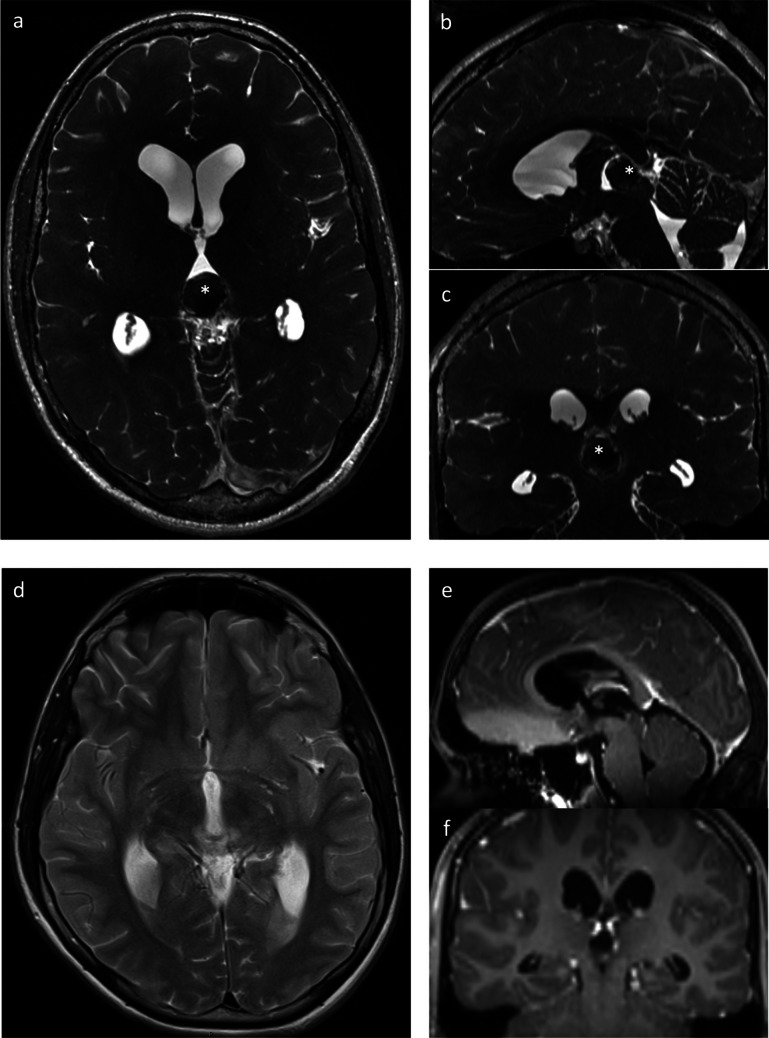
Pre (**a**-**c**) and postoperative (**d**-**f**) MRI imaging of a hemorrhaged pineal cyst (white *) and consecutive aqueduct stenosis with hydrocephalus occlusus removed by microscopic approach via a supracerebellar infrantentorial craniotomy

**Fig. 4:**
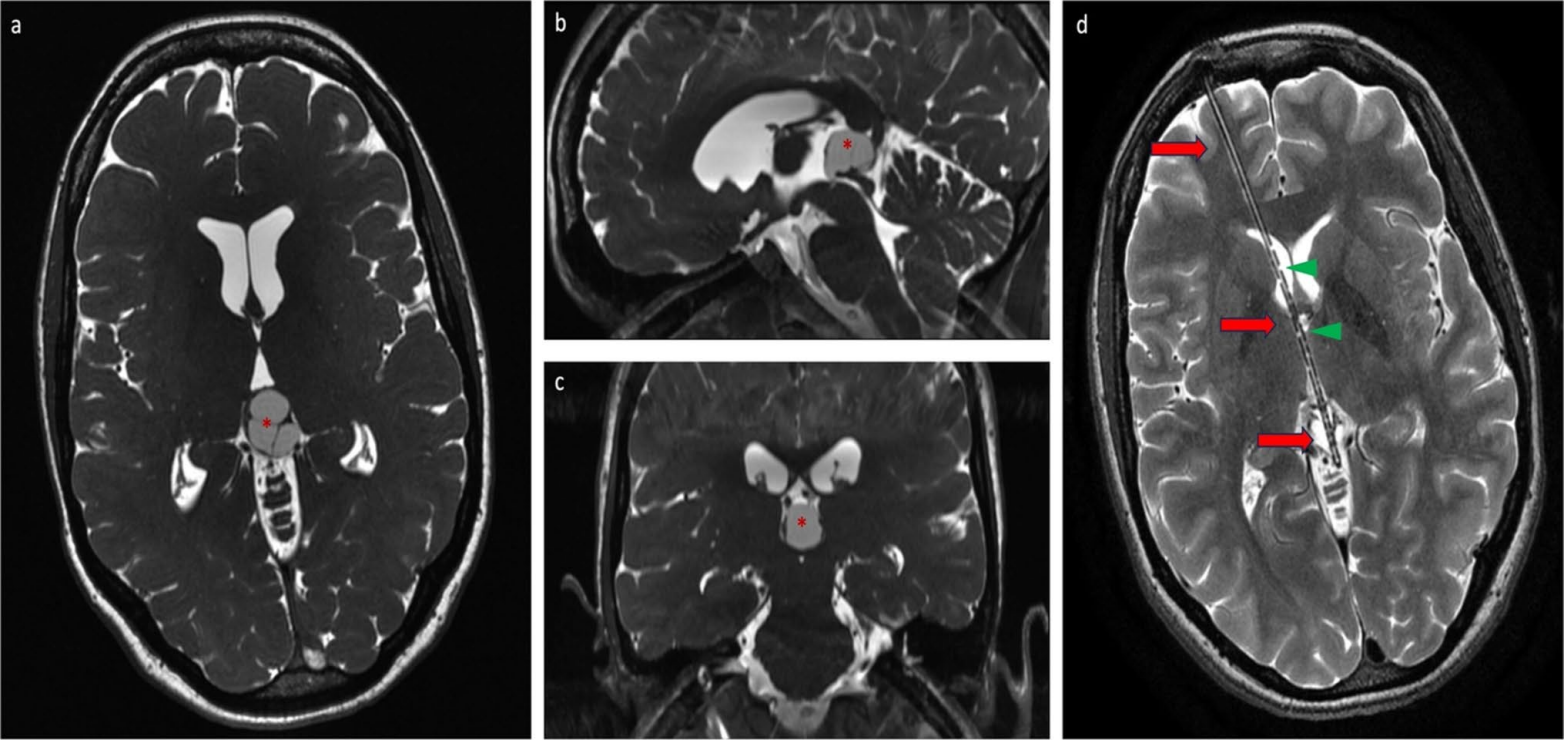
Pre- (**a**-**c**) and postoperative (**d**) MRI imaging of a pineal cyst (red *) and consecutive hydrocephalus occlusus drained through an internal shunt. The course of the catheter is marked by the red arrow. Extra perforations in the catheter to achieve optimal up- and downstream drainage (green arrow head) were added manually. Additionally, a normalized ventricular system can be seen postoperatively

Overall, therapy resulted in a significant reduction in median cyst volume after the first surgery from 4.8 cm^3^ to 1.2 cm^3^ (range: 0–4.8 cm^3^) (*p* < 0.0001). The respective median volumes dropped from 4.0 cm^3^ (range 1.4–9.5 cm^3^) to 0.9 cm^3^ (range 0–4.8 cm^3^) after stereotaxy (*p* < 0.0001), from median 5.5 cm^3^ (range 3.6–19.8 cm^3^) to 1.5 cm^3^ (range 0–4.3 cm^3^) after microsurgery (*p* = 0.02) and from median 6.2 cm^3^ (range 5.1–7.2 cm^3^) to 2.1 cm^3^ (range 0–3.3 cm^3^) after microscopical/endoscopical-assisted cyst fenestration (*p* < 0.0001, Fig. 2a). The extent of the absolute and relative final volume reduction did not differ between the two surgical groups (*p* = 0.1 and *p* = 0.4, respectively; Fig. 2b and c). MR imaging of PCs pre- and postoperatively depending on each surgical approach is shown in Figs. 3 and 4.

The determination of the CTS ratio showed a significant reduction from preoperative mean values of 0.86 ± 0.06 to postoperative 0.23 ± 0.08 (*p* < 0.0001) in PC treated microsurgically/endoscopically. Similarly, the CTS ratios in patients who underwent stereotactic surgery were significant different from preoperative 0.87 ± 0.04 to postoperative 0.48 ± 0.12 (*p* < 0.0001). The comparison between postoperative patients after microsurgery and STX was also statistically significant (*p* = 0.001).

### Measurement of the ventricular width

**Fig. 5:**
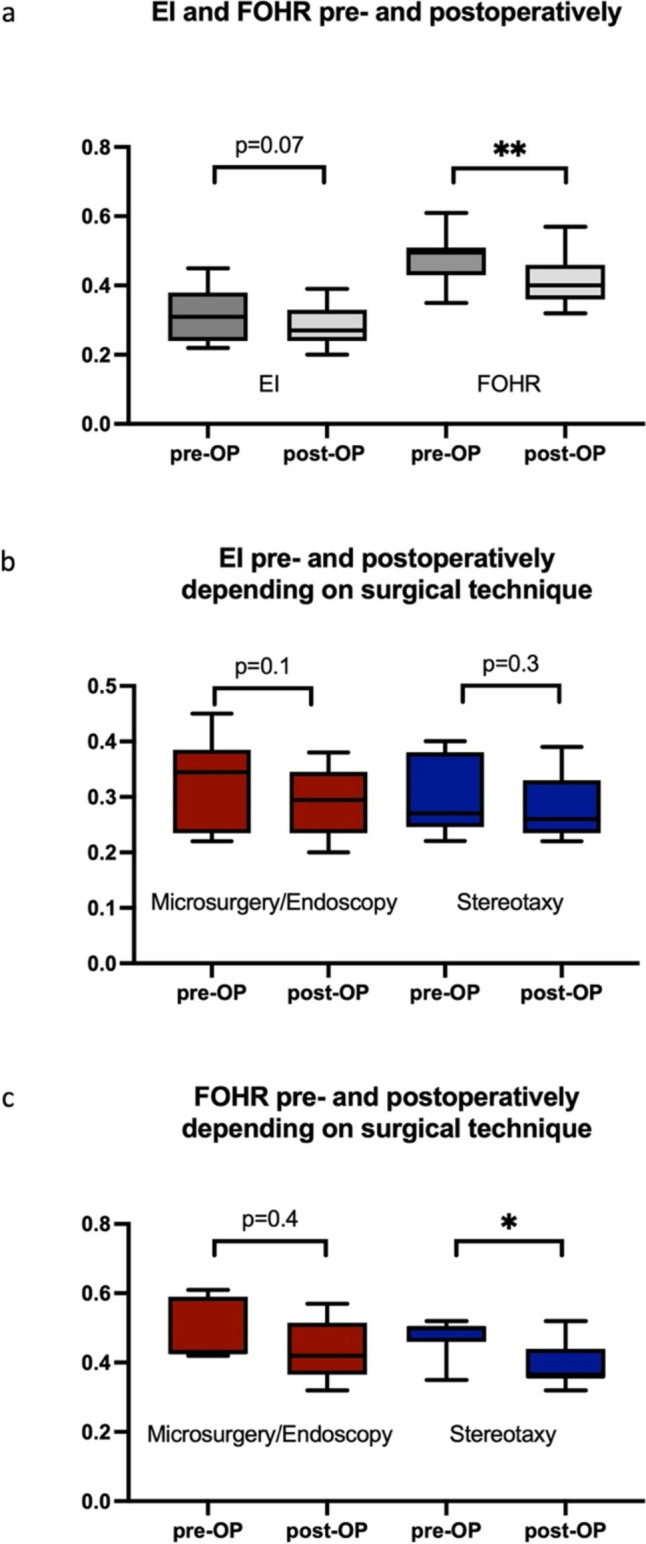
Illustration of the pre- and postoperative EI and FOHR in the overall patient cohort (**a**) and depending on the surgical technique (**b** and **c**). FOHR significantly decreased in the overall cohort (**, *p* = 0.004). The EI showed no significant decrease after stereotactic and microscopic/endoscopic PC fenestration (b). FOHR was significantly reduced after stereotaxy (*, *p* = 0.04) compared to microscopy/endoscopy (*p* = 0.4, c)

The determination of the EI and FOHR in the overall cohort (Fig. 5a) showed a reduction in the median value of the EI from 0.31 to 0.27 (*p* = 0.07) and of the FOHR from 0.49 to 0.4 (*p* = 0.004). However, no significant difference in reduction of the EI could be shown between the stereotactic and microscopy/endoscopy groups (*p* = 0.3 and *p* = 0.1, respectively, Fig. 5b), while FOHR decreased significantly more after stereotactic shunt implantation (*p* = 0.004) compared to microscopy/endoscopy (*p* = 0.4, Fig. 5c).

There was a significant correlation of the preoperative EI with the preoperative (*p* = 0.04, *r* = 0.5) and postoperative (*p* = 0.009, *r* = 0.4) pineal cyst volume, while no significant association was confirmed for the postoperative EI or FOHR.

### Surgical morbidity

Overall, perioperative complications were seen in 3/39 patients (7.7%). Revision surgery was performed in 2 patients due to a CSF leakage after stereotactic catheter implantation. Furthermore, one case of intracystic hemorrhage after stereotaxy was observed, but did not require revision surgery. Complication after microscopy/endoscopy was not seen in our patient cohort. Overall, the rate of complications after stereotaxy and microscopy/endoscopy with subsequent revision surgery did not differ between the treatment modalities (*p* = 0.5).

### Treatment for recurrent cysts

**Fig. 6:**
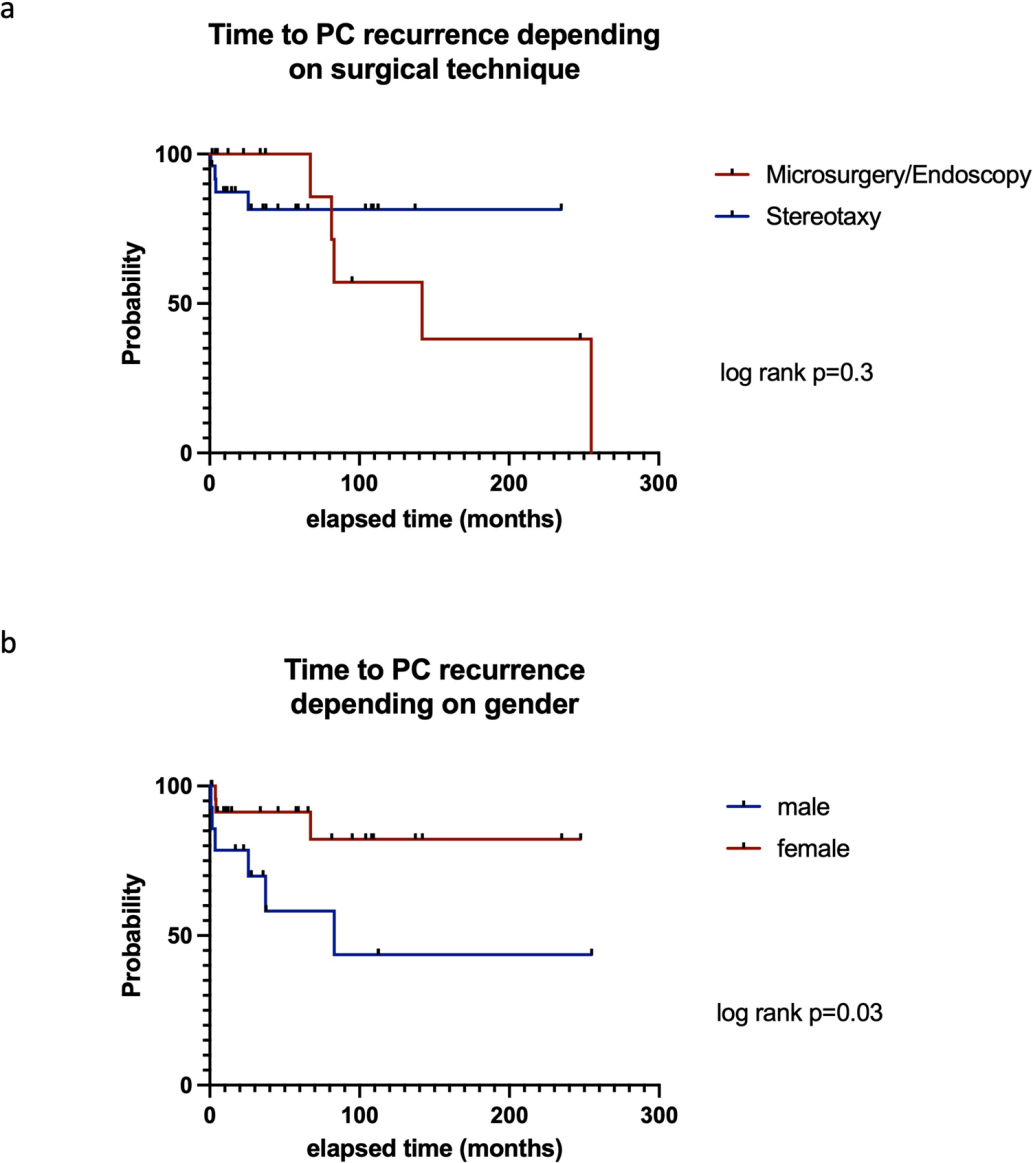
Time to second surgery for recurrent PC after microscopical/endoscopic PC wall fenestration and stereotactic implantation of an internal shunt catheter (Fig. 6a) and based on gender (Fig. 6b)

The median FU for all patients was 58.3 months but was significantly longer in the microscopy/endoscopy group (116.0 ± 96.1 months) compared to the stereotaxy group (64.8 ± 57.1; *p* = 0.04). Overall, local PC recurrence was noted in 9 patients (23.1%). PC recurrences were most often associated with progressive headache (8/9 patients), visual impairment (5/9 patients), epileptic seizures (3/9 patients), increased ICP (2/9 patients), and new paresis or vertigo (each 1/9 patients). Frequency of PC recurrence did not differ according to the initial surgical procedure (5/14 (35.7%) microscopy/endoscopy patients vs. 4/25 (16.0%) stereotaxy patients, *p* = 0.2). The stereotactic recurrences were treated by means of cyst fenestration via a new stereotactic trajectory. This was in all cases a cyst puncture without implantation of a new additional catheter. The initial catheter was left in place and cyst fluid was drained through the additional stereotactic puncture, which did not lead to a further cyst recurrence. Overall time to pineal cyst recurrence (TTR) was 25.2 ± 31.2 months. Median TTR was 3.8 months (range: 0.8–25.8 months) after stereotaxy as compared to 37.3 months (1.6–83.1 months) after microscopical/endoscopic PC fenestration (log rank, *p* = 0.3, Fig. 6a). However, TTR between gender did differ significantly (*p* = 0.03). For details see Fig. 6b. Recurrent PC were treated in 6 cases by stereotactic cyst drainage and in 3 cases by microscopic re-fenestration. A detailed overview of the patients with PC recurrences, their symptoms and the surgical technique used is summarized in Table 3.

**Table 3:** Detailed overview of patients with PC recurrences, their pre- and postoperative symptoms as well as the surgical technique used initially and in the case of recurrence

Patient number	Symptoms		TTR (days)	Symptoms		Surgery 1	Surgery 2	FU (days)
	Pre-OP 1	Post-OP 1		Pre-OP 2	Post-OP 2			
1	epilepsy	none	104	headache, epilepsy	none	stereotaxy	stereotaxy	1727
2	paresis, headache, epilepsy	headache	2016	paresis, headache, epilepsy	none	microsurgery	microsurgery	7972
3	headache, vertigo, increased ICP	none	123	headache, vertigo, increased ICP	none	stereotaxy	stereotaxy	4207
4	headache, visual impairment	none	112	headache, visual impairment	none	microsurgery	microsurgery	5447
5	headache, visual impairment	none	1118	headache, visual impairment	none	microsurgery	stereotaxy	5401
6	headache, epilepsy, visual impairment	none	47	visual impairment, epilepsy, increased ICP	none	microsurgery	microsurgery	295
7	headache	none	773	headache	none	stereotaxy	stereotaxy	1064
8	headache, visual impairment	none	2494	headache, visual impairment	none	microsurgery	stereotaxy	2754
9	headache, visual impairment	none	24	headache, visual impairment	none	stereotaxy	stereotaxy	596

### Prognostic factors for recurrent PC

In univariate analysis, the male gender (*p* = 0.03), a lower Karnofsky performance score (*p* = 0.02), enlarged pre- and postoperative PC volume (*p* = 0.02 and *p* = 0.008, respectively), prolonged surgery time (*p* = 0.04) and an increased preoperative EI (*p* = 0.0005) were associated with PC recurrence. Pineal cyst hemorrhage, an enlarged ventricular system and an elevated FOHR did not influence the recurrence of a pineal cyst significantly. In multivariate analysis the male gender (*p* = 0.01), longer surgery time (*p* = 0.03) and a greater preoperative EI (*p* = 0.007) were confirmed as significant risk factors for cyst recurrence (for details see Table 4).

**Table 4:** Uni- and multivariate analysis for determination of risk factors for recurrent pineal cysts

Variable	odds ratio	95% CI	*p value*
Univariate analysis			
Age	0.2	24.7 – 39.8	0.6
Gender (m vs f)	5.2	0.1 – 0.4	**0.03**
Karnofsky performance score	5.4	88.1 – 93.3	**0.02**
PC hemorrhage	0.9	0.07 – 0.2	0.3
Prominent optic sheaths	0.05	0.2 – 0.6	0.8
PC volume			
Pre-op	6.2	3.5 – 5.9	**0.02**
Post-op	7.9	0.6 – 1.6	**0.008**
Enlarged ventricular system			
Pre-op	1.9	0.2 – 0.6	0.2
Post-op	0.2	−0.03 – 0.2	0.7
Parinaud symptom			
Pre-op	0.8	−0.05 – 0.1	0.8
Post-op	3.6	−0.06 – 0.06	0.07
Incision to suture time	4.1	51.2 – 104.6	**0.04**
Surgical approach			
Microsurgery/endoscopy			
vs. stereotaxy	2.0	0.12 – 0.48	0.2
EI			
Pre-op	14.8	0.2 – 0.5	**0.0005**
Post-op	1.1	0.2 – 0.3	0.3
FOHR			
Pre-op	0.5	0.4 – 0.5	0.6
Post-op	0.3	0.36 – 0.44	0.5
CTS ratio			
Pre-OP	0.04	0.84 – 0.88	0.8
Post-OP	0.08	0.28 – 0.48	0.8
Multivariate analysis			
Gender (m vs f)	2.7	0.06 – 0.4	**0.01**
Karnofsky performance score	1.4	−0.004 – 0.02	0.2
PC volume			
Pre-op	0.4	−0.04 – 0.05	0.7
Post-op	0.1	−0.09 – 0.1	0.9
Incision to suture time	2.3	0.0002 – 0.003	**0.03**
EI pre-op	3.0	0.09 – 0.5	**0.007**

## Discussion

This monocentric study reflects our results in the surgical treatment of symptomatic PC. It should be emphasized that this group represents about 1/3 of the total cohort of patients with PC presenting to our neurosurgical clinic. Since MRI imaging is often performed for nonspecific complaints (such as head and neck pain) and PC is occasionally visible, it is unclear whether small cysts, for example, also cause positional headaches and can therefore be misclassified as asymptomatic. However, these incidental findings were also followed up in our clinical routine and proved to be clinically and imaging stable, so that no surgical intervention was necessary in the further course.

### Clinical findings

The patient characteristics of our study show that females were slightly more frequently prone to have PCs. Similar findings were reported in earlier studies [15, 17, 25, 37, 41]. It is unclear whether there may be a bias here, as headaches occur somewhat more frequently in women and MRI images are performed correspondingly more frequently [4, 15, 19, 25]. The PCs became symptomatic, especially in the 3rd and 4th decade of life. The predominant symptoms included refractory headaches, epileptic seizures, visual impairment and vertigo, which is also in line with previous studies [15, 25, 26]. Notably, almost half of the surgically treated patients showed a preoperative hydrocephalus or prominent optic nerve sheaths.

### Surgical procedures

A particular quality at our center is our proven expertise in both microsurgical operations in the pineal region using various access routes and visualization options (microscope and endoscope) as well as individualized stereotactic procedures with the aim of permanent cyst drainage. According to further studies [15, 23, 25], the supracerebellar infratentorial approach has been confirmed as a legitimate approach for successful microsurgical cyst resection. In our cohort, this was also the second most common approach in 6/14 patients (42.9%). The stereotactic implantation of an internal shunt catheter has been refined and adjusted over the last decades to offer a less invasive surgical approach to sufficiently drain various intracranial (arachnoidal, pineal or tumor) cysts into the ventricles and/or cisterns [21, 27, 29, 33, 34, 38]. In our cohort, the preferred surgical procedure depends on the number and spatial relationship of the cysts in relation to the solid pineal tissue, the deep cerebral veins, the tectum plate and the anatomical configuration of the ventricles and the steep position of the tentorium. The preferred surgical technique was selected for each patient in consensus between experienced neurosurgeons depending on PC morphology, size and individual (general) risk assessment.

### Clinical outcome

Surgery resulted in a significant reduction of preoperative headache symptoms, epileptic seizures and visual impairments. We found a significant reduction of preoperative hydrocephalus as well as dilated optic nerve sheaths in postoperative MR imaging. Similar findings were described before [4, 15, 25, 43]. The volumetric measurements of the cyst size showed a significant reduction in the overall cohort independent of the respective surgical strategy. The EI improved after cyst drainage or fenestration in the overall patient cohort, which has been described before [8, 36]. While there was no difference of EI improvement between both surgical groups, the FOHR particularly showed a significant reduction after stereotactic catheter implantation. The determination of the CTS ratio showed a significant reduction postoperatively in both microsurgically/endoscopically and stereotactically treated patients. The CTS ratio describes the crowding extent of the PC in the pineal recessus and thus represents a MR morphological biomarker for estimating the pressure effect of PC. Microsurgical/endoscopical treatment resulted in a significantly lower CTS ratio compared to stereotaxy. This is also due to the continued presence of a cyst membrane, which only appears to be less tensioned by an internal shunt and cannot be resected as through a microsurgical or endocopical approach. However, we rarely found values above 0.9, which is also consistent with other studies [9–12, 18, 25, 41].

The determination of the CCOS in the overall cohort showed a mean value of 14.8 ± 1.6 (range 10–16), which reflects a good functional outcome after surgery. In particular, only 2 patients had a value of 10, which corresponds to an “impaired” outcome, while the other 37 patients had values between 11 and 16, which implies a “functional” and “excellent” outcome [2]. Our scores reflect similar results from other studies [5, 13]. The patients of the different surgical procedures also had comparable CCOS values, assuming a good and functional similar outcome after microsurgery/endoscopy and stereotaxy.

### Pineal cyst recurrences and revisions

In our study, we were able to identify significant risk factors for the recurrence of symptomatic PC. In multivariate analysis, male gender, the operation time and a preoperatively increased EI were of significant importance. However, we did not find a significant influence of the pre- or postoperative FOHR on PC recurrencies. This has not been reported so far [8, 15]. In conclusion, the presence of hydrocephalus, the EI value as well gender must be taken into account regarding the risk of PC recurrence after microsurgery and stereotaxy.

### Limitations

A major limitation of this retrospective study is the non-randomized use of the available surgical strategies based on a case-based individual decision process by experienced neurosurgeons. Despite the fact that postoperative outcome scales – as here the CCOS – can be used to objectify the functional outcome of patients [2, 13], outcome data could only be drawn from postoperative time points at last follow-up. In addition, the rather short follow-up period of this study must be emphasized. Due to the small patient number, a possible bias cannot be excluded, especially in the multivariate analysis. In this respect, further prospective and even multicenter studies with larger patient cohorts and postoperative standardized evaluation of patients’ outcome may allow for reproducible, non-biased multivariate risk assessment and confirm our retrospective findings.

## Conclusions

This study compares various surgical procedures for the treatment of pineal cysts in a cohort of 39 patients. Microsurgical/endoscopic and stereotactic procedures proved to be safe and effective in volume reduction and improvement of symptoms. The presence of a preoperatively increased EI and the male gender proved to be significant risk factors for recurrent cysts in our study cohort, which may be useful for future decisions on the most suitable surgical strategy by minimizing the need for multiple surgical procedures as well the associated risks of an operation. Nevertheless, the moderate number of patients should be taken into account, drawing significant conclusions only to a limited extent.

## Supplementary Information

Below is the link to the electronic supplementary material.

Supplementary file1 (DOCX 45 KB)

## Data Availability

The datasets used and/or analysed during the current study are available from the corresponding author on reasonable request.
