# Supplementary material for: Comparison of surgical approaches and outcome for symptomatic pineal cysts: microscopic/endoscopic fenestration vs. stereotactic catheter implantation
Source: Acta Neurochir (Wien). 2025 Jan 31;167(1):27. doi: 10.1007/s00701-025-06445-3 (PMC11785698; doi:10.1007/s00701-025-06445-3)
Supplement: Supplementary file 1 — Supplementary file1 (DOCX 45 KB) [file 701_2025_6445_MOESM1_ESM.docx]

| **Patient number** | **Sex**  **1: M**  **0: F** | **KPS** | **Age OP1**  **(yrs)** | **OP1**  **1: micro**  **0: stx** | **Type of microsurgery**  **1: M**  **0: EM** | **Age OP2**  **(yrs)** | **OP2**  **1: micro**  **0: stx** | **Rec.** | **Time to recurrence (days)** | **Number surgeries** | **Incision to suture time (min) OP1** | **Cyst rupture**  **1: yes**  **0: no** | **Cyst hemorrhage**  **1: yes**  **0: no** | **Dilated optic nerve sheaths**  **1: yes**  **0: no** | **Symptoms pre-OP** | **Symptoms post-OP** | **CCOS** | **Symptoms duration pre-OP (months)** | **C pre-OP**  **(mm)** | **TS pre-OP**  **(mm)** | **CTS pre-OP** | **C post-OP**  **(mm)** | **TS post-OP**  **(mm)** | **CTS post-OP** | **FU (days)** | **Hydrocephalus pre-OP**  **1: yes**  **0: no** | **Hydrocephalus post-OP**  **1: yes**  **0: no** | **Parinaud pre-OP**  **1: yes**  **0: no** | **Parinaud post-OP**  **1: yes**  **0: no** |
| --- | --- | --- | --- | --- | --- | --- | --- | --- | --- | --- | --- | --- | --- | --- | --- | --- | --- | --- | --- | --- | --- | --- | --- | --- | --- | --- | --- | --- | --- |
| 1 | 0 | 70 | 51,3 | 1 | 1 | 56,9 | 1 | 1 | 2016 | 2 | 245 | 0 | 0 | 0 | 1 3 4 7 | 3 | 14 |  | 9,5 | 10,7 | 0,887850467 | 5,9 | 14,2 | 0,415492958 | 7972 | 0 | 0 | 0 | 0 |
| 2 | 1 | 90 | 43,9 | 1 | 1 |  |  | 0 |  | 1 | 84 | 0 | 0 | 0 | 3 5 | 5 | 14 |  | 7 | 8 | 0,875 | 4,4 | 10 | 0,44 | 7647 | 0 | 0 | 0 | 0 |
| 3 | 0 | 90 | 62 | 1 | 1 |  |  | 0 |  | 1 | 79 | 0 | 0 | 0 | 3 5 9 | 0 | 14 |  |  |  |  |  |  |  | 4256 | 1 | 1 | 0 | 0 |
| 4 | 1 | 70 | 40,1 | 1 | 1 | 43,2 | 0 | 1 | 1118 | 2 | 259 | 0 | 0 | 1 | 3 9 | 0 | 15 |  | 12 | 14 | 0,857142857 |  |  |  | 5401 | 0 | 0 | 0 | 0 |
| 5 | 0 | 90 | 39,2 | 1 | 1 |  |  | 0 |  | 1 | 68 | 0 | 0 | 0 | 3 | 0 | 16 |  | 8,5 | 10,1 | 0,841584158 | 0 |  | 0 | 2854 | 1 | 0 | 0 | 0 |
| 6 | 0 | 90 | 11,3 | 1 | 1 |  |  | 0 |  | 1 | 234 | 0 | 0 | 0 | 3 | 0 | 16 |  |  |  |  |  |  |  | 2442 | 0 | 0 | 0 | 0 |
| 7 | 0 | 90 | 32,6 | 1 | 1 |  |  | 0 |  | 1 | 78 | 0 | 0 | 1 | 3 9 | 0 | 13 |  | 12,7 | 13,2 | 0,962121212 |  |  |  | 7426 | 1 | 1 | 0 | 0 |
| 8 | 0 | 90 | 46,1 | 0 |  |  |  | 0 |  | 1 | 45 | 0 | 0 | 0 | 3 | 0 | 16 |  | 9,8 | 11,2 | 0,875 | 7,3 | 14 | 0,521428571 | 1729 | 0 | 0 | 0 | 0 |
| 9 | 0 | 100 | 16,3 | 0 |  |  |  | 0 |  | 1 | 47 | 0 | 0 | 0 | 3 5 | 3 5 | 10 |  | 9,6 | 11,1 | 0,864864865 | 4,9 | 13 | 0,376923077 | 7047 | 0 | 0 | 0 | 0 |
| 10 | 1 | 90 | 5,1 | 0 |  | 7,2 | 0 | 1 | 773 | 2 | 46 | 0 | 0 | 0 | 3 | 0 | 16 |  | 8,2 | 9,6 | 0,854166667 | 5 | 11,8 | 0,423728814 | 1064 | 1 | 0 | 0 | 0 |
| 11 | 0 | 90 | 31,3 | 1 | 0 | 31,5 | 1 | 1 | 112 | 2 | 224 | 0 | 0 | 0 | 3 9 | 9 | 13 |  | 11,6 | 13 | 0,892307692 | 6,8 | 15,6 | 0,435897436 | 5447 | 0 | 0 | 1 | 1 |
| 12 | 0 | 100 | 22,5 | 0 |  |  |  | 0 |  | 1 | 39 | 0 | 0 | 0 | 3 | 0 | 16 |  | 8,8 | 9,9 | 0,888888889 | 4,3 | 9,3 | 0,462365591 | 1769 | 0 | 0 | 0 | 0 |
| 13 | 1 | 100 | 19,6 | 0 |  | 19,9 | 0 | 1 | 104 | 2 | 37 | 0 | 0 | 0 | 4 | 0 | 15 |  | 11,4 | 13 | 0,876923077 | 11,5 | 17,3 | 0,664739884 | 1727 | 1 | 0 | 0 | 0 |
| 14 | 1 | 80 | 45,2 | 1 | 0 | 52 | 0 | 1 | 2494 | 2 | 212 | 0 | 0 | 0 | 3 9 | 0 | 11 | 12 | 8,8 | 11,1 | 0,792792793 |  |  |  | 2754 | 1 | 0 | 0 | 0 |
| 15 | 0 | 100 | 37 | 1 | 1 |  |  | 0 |  | 1 | 211 | 0 | 0 | 0 | 3 5 | 3 | 15 |  | 13,8 | 15,3 | 0,901960784 | 3,8 | 10,4 | 0,365384615 | 1015 | 0 | 0 | 0 | 0 |
| 16 | 0 | 90 | 22,6 | 1 | 0 |  |  | 0 |  | 1 | 201 | 0 | 0 | 0 | 3 | 0 | 16 |  | 8,2 | 11 | 0,745454545 | 5,6 | 13,7 | 0,408759124 | 370 | 0 | 0 | 0 | 0 |
| 17 | 0 | 90 | 26,4 | 0 |  |  |  | 0 |  | 1 | 56 | 0 | 0 | 1 | 3 4 7 | 0 | 16 | 0,5 | 16,1 | 17 | 0,947058824 | 9,4 | 15,5 | 0,606451613 | 42 | 1 | 0 | 0 | 0 |
| 18 | 0 | 100 | 18,4 | 0 |  |  |  | 0 |  | 1 | 49 | 0 | 0 | 0 | 3 4 7 | 0 | 14 | 3 | 9 | 10,3 | 0,873786408 |  |  |  | 3242 | 0 | 0 | 0 | 0 |
| 19 | 0 | 90 | 25,2 | 0 |  | 25,5 | 0 | 1 | 123 | 2 | 45 | 0 | 0 | 1 | 3 5 7 | 0 | 16 | 2 | 15 | 17 | 0,882352941 | 8,2 | 15,8 | 0,518987342 | 4207 | 1 | 0 | 0 | 0 |
| 20 | 0 | 100 | 12 | 0 |  |  |  | 0 |  | 1 | 43 | 0 | 0 | 0 | 3 9 | 0 | 16 |  | 14,6 | 16 | 0,9125 | 9,9 | 14,2 | 0,697183099 | 3122 | 0 | 0 | 0 | 0 |
| 21 | 1 | 90 | 18,1 | 0 |  |  |  | 0 |  | 1 | 56 | 0 | 1 | 1 | 3 5 9 | 0 | 16 |  | 15,4 | 18,4 | 0,836956522 | 5,6 | 15 | 0,373333333 | 3378 | 1 | 0 | 0 | 0 |
| 22 | 0 | 100 | 43,6 | 0 |  |  |  | 0 |  | 1 | 43 | 0 | 0 | 1 | 3 | 0 | 16 | 12 | 10,8 | 13 | 0,830769231 |  |  |  | 1965 | 0 | 0 | 0 | 0 |
| 23 | 1 | 90 | 39,1 | 0 |  |  |  | 0 |  | 1 | 57 | 0 | 0 | 0 | 3 9 | 0 | 16 |  | 10,9 | 12 | 0,908333333 | 7,9 | 13,5 | 0,585185185 | 3372 | 0 | 0 | 0 | 0 |
| 24 | 1 | 90 | 24,8 | 0 |  |  |  | 0 |  | 1 | 47 | 0 | 0 | 0 | 4 | 0 | 16 | 3 | 8,5 | 10,4 | 0,817307692 | 7,4 | 13,4 | 0,552238806 | 1067 | 0 | 0 | 0 | 0 |
| 25 | 0 | 80 | 27 | 0 |  |  |  | 0 |  | 1 | 46 | 0 | 0 | 0 | 10 | 0 | 13 |  | 9,5 | 11,2 | 0,848214286 |  |  |  | 436 | 0 | 0 | 0 | 0 |
| 26 | 0 | 90 | 35,5 | 0 |  |  |  | 0 |  | 1 | 43 | 0 | 0 | 1 | 3 4 9 | 0 | 14 | 3 | 13,1 | 15,3 | 0,85620915 | 5 | 12,2 | 0,409836066 | 4118 | 1 | 0 | 0 | 0 |
| 27 | 1 | 80 | 41,8 | 0 |  |  |  | 0 |  | 1 | 58 | 0 | 0 | 1 | 3 9 | 0 | 15 | 10 | 12,4 | 13,6 | 0,911764706 |  |  |  | 833 | 0 | 0 | 0 | 0 |
| 28 | 0 | 90 | 30 | 0 |  |  |  | 0 |  | 2 | 45 | 0 | 0 | 1 | 3 | 0 | 16 |  | 10,7 | 13,2 | 0,810606061 | 5,9 | 15,2 | 0,388157895 | 1368 | 1 | 0 | 0 | 0 |
| 29 | 0 | 90 | 32,1 | 0 |  |  |  | 0 |  | 1 | 55 | 0 | 0 | 0 | 3 | 0 | 16 | 80 | 6,6 | 7,4 | 0,891891892 |  |  |  | 349 | 0 | 0 | 0 | 0 |
| 30 | 0 | 90 | 16,7 | 0 |  |  |  | 0 |  | 1 | 68 | 0 | 0 | 1 | 3 9 | 0 | 13 |  | 8,8 | 10,7 | 0,822429907 | 6,5 | 14,7 | 0,442176871 | 3278 | 0 | 0 | 0 | 0 |
| 31 | 1 | 80 | 63,6 | 0 |  |  |  | 0 |  | 1 | 49 | 0 | 0 | 1 | 3 | 0 | 16 | 30 |  |  |  |  |  |  | 509 | 1 | 0 | 0 | 0 |
| 32 | 0 | 90 | 30,7 | 0 |  |  |  | 0 |  | 1 | 43 | 0 | 1 | 0 | 3 4 | 0 | 16 | 1 | 15,2 | 17,1 | 0,888888889 |  |  |  | 276 | 0 | 0 | 0 | 0 |
| 33 | 1 | 90 | 15,1 | 1 | 0 |  |  | 0 |  | 1 | 278 | 0 | 1 |  | 3 9 | 0 | 15 |  | 14,6 | 16 | 0,9125 | 0 |  | 0 | 674 | 1 | 0 | 1 | 0 |
| 34 | 1 | 80 | 71,8 | 0 |  | 71,9 | 0 | 1 | 24 | 2 | 56 | 0 | 0 | 1 | 3 9 | 0 | 10 | 2 | 9,5 | 10,3 | 0,922330097 |  |  |  | 596 | 1 | 1 | 0 | 0 |
| 35 | 0 | 90 | 54,7 | 0 |  |  |  | 0 |  | 1 | 43 | 0 | 0 | 0 | 3 9 | 0 | 16 |  | 12 | 15 | 0,8 |  |  |  |  | 1 | 0 | 0 | 0 |
| 36 | 1 | 90 | 49,1 | 0 |  |  |  | 0 |  | 1 | 54 | 0 | 0 | 1 | 3 4 9 | 0 | 15 | 2,5 | 19,8 | 24,9 | 0,795180723 | 3,6 | 14,9 | 0,241610738 | 1129 | 1 | 0 | 0 | 0 |
| 37 | 1 | 90 | 41,3 | 1 | 0 | 41,5 | 1 | 1 | 47 | 2 | 78 | 0 | 0 | 1 | 3 4 9 | 0 | 15 |  | 10,9 | 12,5 | 0,872 | 0 |  | 0 | 295 | 1 | 0 | 0 | 0 |
| 38 | 0 | 80 | 65,8 | 1 | 0 |  |  | 0 |  | 1 | 79 | 0 | 0 | 1 | 3 9 | 0 | 15 | 7 | 11,8 | 13,2 | 0,893939394 | 0 |  | 0 | 150 | 1 | 0 | 0 | 0 |
| 39 | 0 | 90 | 34,3 | 0 |  |  |  | 0 |  | 1 | 39 | 0 | 0 | 0 | 3 | 0 | 16 | 18 | 6,5 | 7,2 | 0,902777778 | 5,2 | 13,3 | 0,390977444 | 26 | 0 | 0 | 0 | 0 |

Sex:

M: male

F: female

Type of microsurgery:

1: microsurgery

0: endoscopic-assistend microsurgery

Rec.: cyst recurrence

KPS: Karnfosky performance score

Symptoms:

0: none

1: paresis

2: sensory disturbance

3: headache

4: epileptic seizures

5: vertigo

6: coordination disturbance

7: increased ICP

8: cranial nerve symptoms

9: visual impairment

10: mnestic disorder

CCOS: Chicao Chiari Outcome Score

C: anteroposterior diameter of cyst

TS: distance tectum – splenium of the corpus callosum

CTS: cyst-tectum-splenium ratio

FU: follow-up
